# Variations of QRS Morphology in Patients with Dilated Cardiomyopathy; Clinical and Prognostic Implications

**DOI:** 10.5681/jcvtr.2014.019

**Published:** 2014-06-30

**Authors:** Taylan Akgun, Sedat Kalkan, Mustafa Kursat Tigen

**Affiliations:** ^1^Kartal Kosuyolu Heart & Research Hospital, Department of Cardiology, Istanbul, Turkey; ^2^Marmara University, Faculty of Medicine, Department of Cardiology, Istanbul, Turkey

**Keywords:** Fragmented QRS, Bundle Branch Block, Idiopathic Dilated Cardiomyopathy

## Abstract

The QRS represents the simultaneous activation of the right and left ventricles, although most of the QRS waveform is derived from the larger left ventricular musculature. Although normal QRS duration is <100 millisecond (ms), its duration and shape are quite variable from patient to patient in idiopathic dilated cardiomyopathy (IDCM). Prolongation of QRS occurs in 14% to 47% of heart failure (HF) patients. Left bundle branch block (LBBB) is far more common than right bundle branch block (RBBB). Dyssynchronous left ventricular activation due to LBBB and other intraventricular conduction blocks provides the rationale for the use of cardiac resynchronization therapy with biventricular pacing in patients with IDCM. Fragmented QRS (fQRS) is a marker of depolarization abnormality and present in significant number of the patients with IDCM and narrow QRS complexes. It is associated with arrhythmic events and intraventricular dyssynchrony. The purpose of this manuscript is to present an overview on some clinical, echocardiographic and prognostic implications of various QRS morphologies in patients with IDCM.

## Introduction

The duration of the QRS complex is normally 0.06 to 0.1 seconds.  This relatively short duration indicates that ventricular depolarization normally occurs very rapidly. If the QRS complex is prolonged (>0.1 sec), conduction is impaired within the ventricles. Moreover, not only the QRS duration but also the shape of the QRS complex is also important. The shape will change when there is abnormal conduction of electrical impulses within the ventricles. Prolongation of QRS (120 ms) occurs in 14% to 47% of heart failure (HF) patients.^1^ LBBB is far more common than RBBB. Fragmented QRS (fQRS) is also present in 23-75% of the patients with idiopathic dilated cardiomyopathy (IDCM) and narrow QRS complexes.^2-6^ The purpose of this manuscript is to present an overview on some clinical, echocardiographic and prognostic implications of various QRS morphologies in patients with IDCM

## Importance of bundle branch blocks and nonspecific intraventricular conduction block in patients with IDCM

LBBB is generally associated with a poorer prognosis in comparison to normal intraventricular conduction, but also in comparison to RBBB .The electrocardiographic criteria for LBBB are outlined in Table 1. LBBB may induce abnormalities in left ventricular performance due to abnormal asynchronous contraction patterns. Asynchronous electrical activation of the ventricles causes regional differences in workload that may lead to left ventricular dilatation, especially due to increased wall mass in late-activated regions.^7^ The adverse effect of ventricular dyssynchrony due to LBBB is more pronounced in the presence of heart failure. Although it was concluded in a paper that presence of LBBB at the baseline is not an independent marker of poorer survival and development of LBBB in patients who are already on treatment with ACE inhibitors and beta-blockers is associated with an adverse outcome^8^, several studies have reported that LBBB is an independent risk factor for mortality in patients with heart failure and is associated with increased all-cause mortality and sudden death at one year.^9-11^ While LBBB was showed as an independent prognostic marker in most studies, the evidence for RBBB is less clear. Cinca et al. showed in their study that patients with RBBB presented with overt signs of right and left HF, more depressed RV motion at echocardiography and more frequently reported a history of coronary heart disease.^12^ Left ventricular ejection fraction (LVEF) was lower in LBBB than in RBBB in an another study.^13^ There is need for more evidence regarding the prognostic role of RBBB in patients with IDCM.

**Table 1:** Old and new “strict LBBB” criteria

A task force from the American Heart Association, the American College of Cardiology, and the Heart Rhythm Society has defined the electrocardiographic features of LBBB
•QRS duration greater than or equal to 120 ms in adults, greater than 100 ms in children 4 to 16 years of age, and greater than 90 ms in children less than four years of age •Broad notched or slurred R wave in leads I, aVL, V5, and V6 and an occasional RS pattern in V5 and V6 attributed to displaced transition of QRS complex •Absent q waves in leads I, V5, and V6, but in the lead aVL, a narrow q wave may be present in the absence of myocardial pathology •R peak time greater than 60 ms in leads V5 and V6 but normal in leads V1, V2, and V3, when small initial r waves can be discerned in the above leads.
New “strict LBBB” criteria proposed by Strauss et al.
•a QRS duration ³130 ms in women or ³140 ms in men, •rS or QS morphology in lead V1 and •mid- QRS notching/slurring in at least 2 of the leads V1, V2, V5, V6, I, or aVL

Electrophysiological studies for assessment of ventricular tachycardia (VT) inducibility were studied in 777 patients (The percentage of patients with heart failure is unknown). Sustained monomorphic VT was induced in 49% and 23% (p ≤0.0001) of patients with QRS ≥120 ms and ≤120 ms, respectively. QRS duration was showed to be an independent risk factor for VT inducibility in multivariate analysis. In fact, the risk of inducible sustained monomorphic VT increased by 2.4% for each 1 ms increase in QRS duration.^14^

Dyssynchronous left ventricular activation due to LBBB and other intraventricular conduction blocks provides the rationale for the use of cardiac resynchronization therapy with biventricular pacing in patients with IDCM. Initial clinical trials for cardiac resynchronization therapy (CRT) enrolled patients with heart failure, a QRS duration of ≥120 ms regardless of the type of conduction abnormality and LV ejection fraction (LVEF) of ≤35%. But given the radically different left ventricular activation sequences in different conduction abnormalities, the response to biventricular pacing is likely to vary substantially. The goal of cardiac resynchronization therapy (CRT) using biventricular pacing is to improve global left ventricular (LV) function by synchronizing activation of the interventricular septum with that of the LV lateral wall.

A meta-analysis was performed to evaluate the effect of CRT on clinical events with regards to different types of baseline conduction abnormalities using data from randomized controlled trials. Four randomized trials totaling 5,356 patients met the inclusion criteria and they concluded that while CRT was very effective in reducing clinical events in patients with LBBB, it did not reduce such events in patients with wide QRS due to other conduction abnormalities.^15^ In the Comparison of Medical Therapy, Pacing, and Defibrillation in Heart Failure (COMPANION) trial, patients without LBBB did not have a statistically significant benefit, and those with QRS duration ≤147 ms had absolutely no benefit.^16^ In the Multicenter Automatic Defibrillator Implantation Trial– Cardiac Resynchronization Therapy (MADIT-CRT) trial, patients with QRS durations ≤150 ms received no benefit.^17^Analysis from prolonged follow-up in MADIT- CRT found that CRT caused a statistically significant increase in heart failure events or mortality in patients without LBBB.^18^ A recent report on 14,946 Medicare patients receiving CRT showed that those without LBBB had significantly increased early and late mortality compared to patients with LBBB, and QRS duration ≥150 ms predicted more favorable outcomes in LBBB but not in RBBB.^19^

The success of CRT in patients with LBBB and longer QRS duration has led to renewed interest in defining LBBB. Recently, Strauss et al reviewed the evidence for LBBB criteria and proposed “strict LBBB” criteria that require a QRS duration ≥130 ms in women or ≥140 ms in men, and also rS or QS morphology in lead V1 and mid- QRS notching/slurring in at least 2 of the leads V1, V2, V5, V6, I, or aVL.^20^ (Table 1).

The increase in QRS duration threshold beyond the conventional criteria of 120 ms or more is because QRS duration should increase by 60 ms with the onset of complete LBBB (40 ms to reach the LV endocardium and 20 additional milliseconds beyond normal to reenter the LV Purkinje network and proceed to the LV lateral wall). This finding came from endocardial mapping study by Auricciho et al.^21^ Grant and Dodge also stated that the average QRS duration prolongation with the onset of supposed LBBB was 50 to 60 ms.^22^ A very recent study by Mascioli et al did investigate the ability of the “strict LBBB” criteria to predict benefit from CRT. In multivariable analysis, the presence of “false LBBB” (meeting conventional LBBB criteria, but not the strict LBBB criteria) predicted a 4-fold increase in heart failure hospitalization or death compared with “true LBBB,” and “true LBBB” was the only variable significantly related to a greater increase in LV ejection fraction (HR, 4.57).^23^ As stated in European Society of Cardiology practice guideline, the low number of HF patients with non-LBBB configuration included in randomized, controlled trials precludes firm conclusions for CRT implantation in this subgroup of patients. The evidence of benefit in patients with non-LBBB configuration is weak, particularly in patients with QRS ≤150 ms and NYHA classes I and II.^24^

## Importance of fQRS in IDCM

fQRS on a routine 12-lead electrocardiogram is a marker of depolarization abnormality. Regions of myocardial scar may produce slow and disorganized conduction and the QRS morphology in the leads overlying scar may be altered and prolonged. Das et al. defined fQRS as the QRS complexes with the presence of an additional R wave (R’) or notching in the nadir of the R wave or the S wave, or the presence of >1 R’ (fragmentation) in 2 contiguous leads.^25^ fQRS was originally defined as narrow QRS complex duration (<120 ms). Das et al. added additional criteria in wide QRS complex: the QRS complex with >2 R’ waves or notches in the R or S wave in a wide QRS complex (BBB, paced QRS or premature ventricular complexes) in 2 contiguous leads. If the QRS complex of PVC only has 2 notches in the R waves, they considered the QRS complex to be fQRS-positive when the notches were more then 40 ms apart and present in 2 contiguous leads.^26^

Although it was more commonly studied in patients with coronary artery disease (CAD), it is not specific for CAD and is also encountered in other myocardial diseases such as congenital heart disease and cardiomyopathy.^27,28^ The correlation between fQRS and ischemic/non-ischemic cardiomyopathy was investigated in some studies. fQRS was present in 23-75% of the patients with IDCM and narrow QRS complexes.^2-6^

Although several studies have shown that fQRS on a routine 12-lead electrocardiogram were associated with increased mortality and arrhythmic events in patients with coronary artery disease^29^, there is relatively little data available regarding IDCM. Jing et al showed in their study that the combined end point of all-cause mortality and ventricular tachyarrhythmias in patients with fQRS increased significantly compared to without fQRS (23.5% vs. 3.4%, P= 0.043) in patients with IDCM during follow-up of 14 ± 5 months. The study showed that f-QRS predicted ventricular tachyarrhythmias but not all-cause mortality. That may be because arrhythmic deaths were prevented by the ICD therapy.^2^ Das et al conducted a study to determine whether fQRS was associated with increased ventricular arrhythmic event and mortality in patients with CAD and IDCM^6^ fQRS on 12-lead ECG was found to be a predictor of arrhythmic events in patients with CAD and DCM. fQRS is associated with a significantly decreased time to first arrhythmic event compared with non-fQRS and wide QRS. Nevertheless, In a prospective, multisite cohort of 842 patients with left ventricular dysfunction (ejection fraction < or =35%) representing both ischemic and nonischemic etiology, the association between fQRS and all-cause and arrhythmic mortality was evaluated overall and stratified by ICD status. Rates of all-cause mortality did not differ between the fQRS+ (19.7%) and fQRS- (24.1%) groups; (adjusted hazard ratio, 0.88; 95% confidence interval, 0.63-1.22; P=0.43) Additionally, rates of arrhythmic mortality were similar between the fQRS+ (9.9%) and fQRS- (12.7%) groups (adjusted hazard ratio, 0.77; 95% confidence interval, 0.49-1.31; P= 0.38). They concluded that these findings do not provide evidence that fQRS would be effective in risk stratifying patients eligible for ICD therapy for primary prevention.^30^

fQRS was also showed to be associated with significant intraventricular dyssynchrony in patients with nonischemic cardiomyopathy, narrow QRS and sinus rhythm.^4^ Tigen et al included 60 patients with IDCM in their study. Forty patients had a fragmented QRS in their baseline ECG, and 20 patients did not have a fragmented QRS. Patients were analyzed for correlation between fragmented QRS complexes and intraventricular dyssynchrony. They concluded that fragmentation in the resting ECG is associated with significant intraventricular dyssynchrony in patients with nonischemic cardiomyopathy, narrow QRS and sinus rhythm. Their study also showed that among dyssynchronic patients, the fragmented ECG segment has a high sensitivity (75.8%) and specificity (76%) to locate the maximal dyssynchronic segment or one of the dyssynchronic segments. Yusuf et al performed a study to assess the sensitivity, specificity and positive predictive value of fQRS complex on the surface ECG to detect significant intraventricular dyssynchrony in symptomatic patients of IDCM.^31^ They showed that fQRS is a marker of electrical dyssynchrony, localizes the dyssynchronic segment and might be useful in identifying patients who can benefit from cardiac resynchronization therapy. These findings bring to mind the question whether the fQRS is a useful predictor in identifying patients who can benefit from CRT. However, current guidelines have no recommendation for CRT in HF patients having fQRS on their ECG.^24^

## Conclusion

Left bundle branch block (LBBB) is generally associated with a poorer prognosis in comparison to normal intraventricular conduction, but also in comparison to right bundle branch block. Some CRT trials emphasize the importance of accurately defining LBBB. New criteria were proposed to identify patients with LBBB who are most likely to benefit from CRT. It is seen that new “strict LBBB” criteria have increased the specificity of complete LBBB diagnosis in the presence of LV dilatation and incomplete LBBB, which is critical for selecting CRT patients. CRT non-response rate is higher in patients with RBBB and nonspecific intraventricular conduction delay. fQRS is a marker of depolarization abnormality and present in significant number of the patients with IDCM. It is associated with arrhythmic events and intraventricular dyssynchrony. Although it is more extensively studied in coronary artery disease, it may be valuable in arrhythmic risk stratifying of patients with IDCM. Using fQRS in selection of patients with HF for CRT needs to be studied through randomized trials.

## Ethical issues

Not applicable.

## Competing interests

Authors declare no conflict of interest in this study.
